# The reference value of anti-Müllerian hormone to diagnose polycystic ovary syndrome is inversely associated with BMI: a retrospective study

**DOI:** 10.1186/s12958-023-01064-y

**Published:** 2023-02-01

**Authors:** Menghui Zhang, Xiaocong Liu, Xiaolu Xu, Jing Li, Zhiqin Bu, Qingling Yang, Hao Shi, Wenbin Niu, Shanjun Dai, Yuling Liang, Yihong Guo

**Affiliations:** 1 Reproductive Medicine Centre, First Affiliated Hospital of Zhengzhou University, Zhengzhou, 450052 China; 2 Henan Key Laboratory of Reproduction and Genetics, First Affiliated Hospital of Zhengzhou University, Zhengzhou, 450052 China

**Keywords:** BMI, AMH, PCOS, Diagnosis, Ovulatory dysfunction

## Abstract

**Background:**

This study aimed to evaluate the cut-off value of anti-Müllerian hormone (AMH) combined with body mass index (BMI) in the diagnosis of polycystic ovary syndrome (PCOS) and polycystic ovary morphology (PCOM).

**Methods:**

This retrospective study included 15,970 patients: 3775 women with PCOS, 2879 women with PCOM, and 9316 patients as controls. Multivariate logistic regression analysis was used to calculate adjusted odds ratios (ORs) and 95% confidence intervals (CIs) for AMH. We randomly divided the patients into two data sets. In dataset 1, a receiver operating characteristic (ROC) curve was generated to analyze the accuracy of basic AMH levels in diagnosing PCOS and PCOM. The optimal cut-off value was calculated in dataset 1 and validated in dataset 2, expressed as sensitivity and specificity.

**Results:**

In the PCOS group, obese patients had the lowest AMH levels, while underweight patients had the highest AMH level (*P* < 0.001). After adjusting for age, the ratio of luteinizing hormone (LH) and follicle stimulating hormone (FSH), serum testosterone level, and BMI, AMH was an independent predictor of PCOS and PCOM. In the group with BMI < 18.5 kg/m^2^, the optimistic AMH cut-off value was 5.145 ng/mL with a sensitivity of 84.3% and specificity of 89.1%, whereas in the BMI ≥ 28 kg/m^2^ group, the optimistic AMH cut-off value was 3.165 ng/mL with a sensitivity of 88.7% and specificity of 74.6%. For the BMI range categories of 18.5–24, 24.0–28 kg/m^2^, the optimistic AMH cut-off values were 4.345 ng/mL and 4.115 ng/mL, respectively. The tendency that the group with lower weight corresponded to higher AMH cut-off values was also applicable to PCOM. In the same BMI category, patients with PCOM had a lower AMH diagnosis threshold than those with PCOS (< 18.5 kg/m^2^, 5.145 vs. 4.3 ng/mL; 18.5–24 kg/m^2^, 4.345 vs. 3.635 ng/mL; 24.0–28 kg/m^2^, 4.115 vs. 3.73 ng/mL; ≥ 28 kg /m^2^, 3.165 vs. 3.155 ng/mL). These cut-off values had a good diagnostic efficacy in the validation dataset. Based on different phenotypes and severity of ovulation disorders, the distribution of AMH in PCOS were also significantly different (*P* < 0.001).

**Conclusions:**

AMH is a potential diagnostic indicator of PCOS and is adversely associated with BMI. The AMH cut-off value for diagnosing PCOS was significantly higher than that for PCOM.

**Supplementary Information:**

The online version contains supplementary material available at 10.1186/s12958-023-01064-y.

## Background

Polycystic ovary syndrome (PCOS) is a highly heterogeneous disease that presents as an alternative combination of polycystic ovary morphology (PCOM), ovulation disorder, hyperandrogenism, and other metabolic and psychological disorders [1, 2]. As one of the most common reproductive diseases affecting reproductive-aged women, the worldwide prevalence of PCOS varies from 5 to 20% based on ethnicity, diagnostic criteria, and epidemiological methodology [3, 4].

Anti-Müllerian hormone (AMH), an ovarian granulosa cell-specific member of the transforming growth factor β (TGF-β) family, is regarded as an ideal indicator of ovarian reserve in populations with different diseases and ages [5, 6] and is an accurate predictor of the response and outcomes of assisted reproductive technology (ART) treatment [7, 8]. AMH is believed to be a surrogate indicator of antral follicle count (AFC) for the diagnosis of PCOS, although there is no consensus on a general diagnostic threshold because of the use of different testing methods and populations included in different studies [9, 10]. In addition, the heterogeneity of patients with PCOS has seldom been considered when calculating AMH cut-off values.

The incidence of obesity, which is normally assessed according to body mass index (BMI), is higher in the PCOS population than in the non-PCOS population [11]. Although a consensus has not yet been reached, previous studies have indicated an inverse relationship between serum AMH levels and BMIs in women with PCOS and in the general population [12]. A previous study constructed a patient-specific model that combined AMH levels and BMI to predict oligo-anovulation disorders [13]. However, it is not clear whether the AMH cutoff value for predicting PCOM, as a characteristic of PCOS, is different from that of PCOS.

Thus, in this study, we aimed to explore the predictive power of AMH in infertile women with PCOS/PCOM and determine the BMI-stratified AMH thresholds for diagnosing PCOS/PCOM.

## Methods

### Participants

This cross-sectional study that included 22,965 Chinese infertile women who visited the hospital between January 2016 and June 2021. All participants were under 35 years of age. All data were acquired from the clinical reproductive medicine management system/electronic medical record cohort database (CCRM/EMRCD) at the Reproductive Medicine Centre of The First Affiliated Hospital of Zhengzhou University in China. The included patients with PCOS were diagnosed according to the modified Rotterdam criteria, which included menstrual abnormalities (menstrual cycle > 35 days) combined with either hyperandrogenism or PCOM [14]. PCOM is diagnosed when 12 or more follicles measuring 2–9 mm in diameter are observed on transvaginal sonography (TVS) is observed. Women less than 21 years of age, with chromosomal abnormalities, diminished ovarian reserve, ovarian abnormalities, and patients whose data were not fully documented were excluded. Some diseases leading to hyperandrogenism and ovulation dysfunction,including tumors, congenital adrenal hyperplasia, amenorrhea, hyperprolactinemia, and thyroid dysfunction, were ruled out. Finally, 15,970 women between 21 and 35 years of age were included in the study.

The "PCOM" group included PCOM in the ovaries with regular cycles and without hyperandrogenism. The control group consisted of patients without PCOM with regular cycles and without hyperandrogenism. All participants were randomized to either the development or validation data set, and the ratio of the group sizes was 2:1. This study was approved by the Institutional Review Board and Ethics Committee of the First Affiliated Hospital of Zhengzhou University (grant no. 2017-KY-15). Informed consent was obtained from all participants.

### Biochemical assays and follicle count

All serum measurements were performed at the Endocrine Laboratory of the Reproductive Medicine Center. When endometrial thickness was less than 5 mm, no dominant follicle growth and diameter of follicles < 5 mm in the ovaries, or during the second to fourth days of menstruation, blood samples for AMH and basic hormones including follicle stimulating hormone (FSH), luteinizing hormone (LH), testosterone (T), estrogen (E2), prolactin, and progesterone (P) were drawn for analysis before ovarian stimulation. Samples were stored at -20 °C until assayed. Hormone levels were measured using a fully automated Elecsys® immunoassay analyzer (Roche, Germany). On the second to seventh days after menstruation ended, an ultrasound technician used a Voluson S8 color Doppler transvaginal ultrasound diagnostic instruments (GE, United States) to monitor the ovary, including antral follicle count.

### Statistical analysis

Statistical analysis was performed using SPSS 26.0 (IBM Corp., USA). Continuous variables are presented as mean values and corresponding standard deviations. We use univariate analysis of variance (ANOVA) to compare continuous variables between groups, and further pairwise post-hoc analysis was performed for statistically significant changes. The LSD test was used for homogeneity of variance data, and the Tamhane S T2 test was used for data that did not meet the homogeneity of variance. Multivariate logistic regression analysis was used to calculate adjusted odds ratios (ORs) and 95% confidence intervals (CIs) for AMH. Multivariate analyses were adjusted for age, BMI, basic serum T, basic LH, and FSH levels. We randomly divided the patients into two data sets. The random seed was set to 2,000,000, and the function rv.UNIFORM(0,1) was used to generate random numbers. Based on the percentile, random numbers were divided equally into three groups using visual bins. The first two groups were named dataset 1, and the last group was named dataset 2. In dataset 1, a receiver operating characteristic (ROC) curve was generated to analyze the diagnostic accuracy of basic AMH levels in diagnosing PCOS and PCOM, which was expressed as the area under the curve (AUC). The optimal cutoff value was determined based on the Youden index. When there were multiple maximum values of the Youden index, the value with the highest specificity was selected as the optimal cut-off value. These tests were also performed for the BMI subgroup. The diagnostic efficacy of the best cut-off value was validated in dataset 2 and expressed as sensitivity and specificity. All tests were two-sided, and *P* value < 0.05 were considered statistically significant.

We used GraphPad Prism for Windows (version 8.3.0, GraphPad Software, San Diego, California, USA; www.graphpad.com) to generate ROC curves. We used the R Package, Version 4.1.2 (The R Foundation, Vienna, Austria) to draw the violin diagram.

## Results

### Patient characteristics

**Fig. 1:**
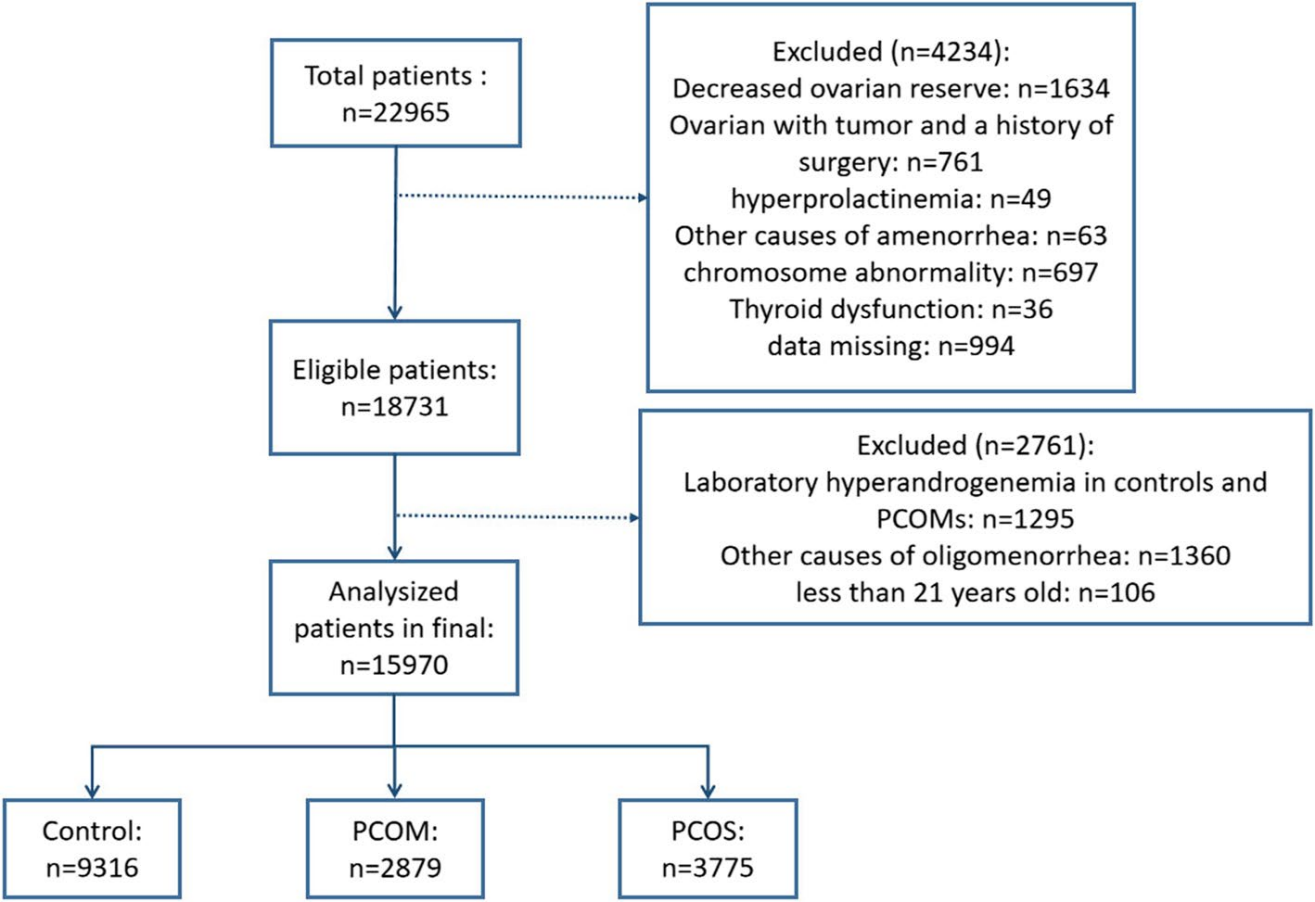
The diagram of study participants. PCOS, polycystic ovary syndrome; PCOM, polycystic ovary morphology

The study ultimately involved 15,970 patients, including 3775 women with PCOS, 2879 women with PCOM, and 9316 controls (Fig. 1). All participants were aged between 21 and 35 years. As shown in Table 1, patients with PCOS and PCOM were younger than controls (*P* < 0.001). However, there was no significant difference in age between the patients with PCOS and their PCOM counterparts. As expected, patients with PCOS had the highest BMI and serum AMH level (*P* < 0.001). In addition, the LH/FSH ratio was higher in women with PCOS than in women with PCOM and the control groups ( 1.68 vs 0.98 vs 0.77, *P* < 0.001, respectively).

**Table 1:** Basic clinical characteristics of Control, PCOM, and PCOS groups

	**Control (** ***n*** ** = 9316)**	**PCOM (** ***n*** ** = 2879)**	**PCOS (** ***n*** ** = 3775)**	***P*** ** value**
Age (y)	29.19 ± 2.98^2,3^	28.06 ± 3.079^1^	28.05 ± 3.073^1^	< 0.001*
BMI (kg/m^2^)	22.36 ± 3.06^2,3^	22.85 ± 3.31^1,3^	24.34 ± 3.58^1,2^	< 0.001*
T (ng/ml)	0.24 ± 0.10^2,3^	0.28 ± 0.10^1,3^	1.18 ± 6.19^1,2^	< 0.001*
AMH (ng/ml)	2.92 ± 1.55^2,3^	5.27 ± 2.55^1,3^	7.55 ± 4.00^1,2^	< 0.001*
LH/FSH	0.77 ± 0.40^2,3^	0.98 ± 0.55^1,3^	1.68 ± 1.12^1,2^	< 0.001*

Data were presented as the mean ± SD. *PCOS* Polycystic ovary syndrome, *PCOM* Polycystic ovary morphology, *BMI* Body mass index, *T* Testosterone, *AMH* Anti-Müllerian hormone, *FSH* Follicle stimulating hormone, *LH* Luteinizing hormone

^*^P: significant difference among the groups. ^1^*P*: significant difference from the control group; ^2^*P*: significant difference from the PCOM group; ^3^*P*: significant difference from the PCOS group

We divided the patients into four groups as the underweight group (BMI < 18.5 kg/m^2^), normal weight group (18.5 kg/m^2^ ≤ BMI < 24 kg/m^2^), overweight group (24 kg/m^2^ ≤ BMI < 28 kg/m^2^) and obese group (BMI ≥ 28 kg/m^2^) according to guidelines based on the Chinese population [15]. Our results showed that the obese group had the lowest AMH levels, and the underweight group had the highest AMH levels (*P* < *0.001*) (Supplementary Fig. 1).

### Logistic regression analysis for the diagnosis of PCOS and PCOM

Multiple logistic regression analysis was used to evaluate the relationship between serum AMH levels, PCOS, and PCOM. After adjusting for age, LH/FSH ratio, serum T level, and BMI, AMH was a separate predictor of PCOS and PCOM (PCOM: OR 1.839, 95%CI 1.788–1.892, *P* = 0.000; PCOS: OR 2.085, 95% CI 2.022–2.150, *P* = 0.000) (Table 2).

**Table 2:** The correlation between AMH and PCOS/PCOM

	**AMH (ng/mL)**	***P***	^**a**^**Adjusted OR (95% CI)**
PCOM	5.27 ± 2.55	.000	1.839 (1.788–1.892)
PCOS	7.55 ± 4.00	.000	2.085 (2.022–2.150)

The reference is the control group

^a^Adjusted for age, BMI, LH/FSH, T. *PCOS* Polycystic ovary syndrome, *PCOM* Polycystic ovary morphology, *AMH* Anti-Müllerian hormone

### ROC analysis of serum AMH thresholds based on different BMIs

**Fig. 2:**
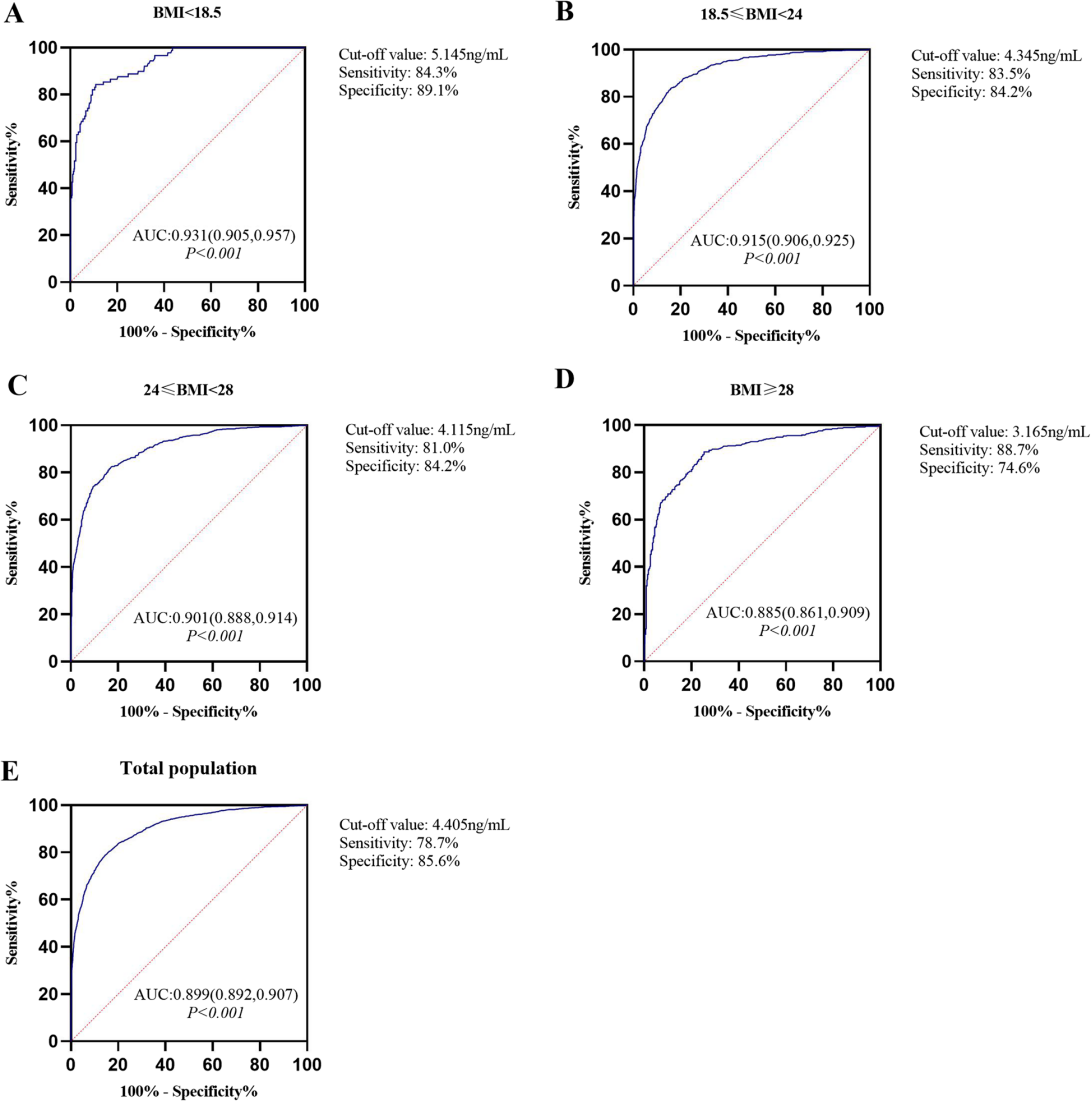
ROC curves for the diagnosis of PCOS based on different BMIs. AUC, area under the curve; BMI, body mass index

We randomly divided the total population into two datasets in a 2:1 ratio. The number of participants in Dataset 1 was 10,645, including 6245 patients in the control group, 1913 patients in the PCOM group, and 2487 patients in the PCOS group. The number of participants in dataset 2 was 5325, including 3071 patients in the control group, 966 patients in the PCOM group, and 1288 patients in the PCOS group. To clarify the optimal AMH threshold for BMI stratification, different subgroups were divided according to BMI level: total population, underweight group, normal weight group, overweight group, and obese group. ROC analysis was performed on dataset 1 (Fig. 2). The cut-off values and AMH diagnostic efficiencies are listed in Table 3 and Supplementary Table 2.

**Table 3:** Results from the ROC analysis for the diagnosis of PCOS based on different BMIs

BMI Groups	AUC	*P* value	The optimal cut-off value of AMH (ng/ml)	Sensitivity (%)	Specificity (%)
BMI < 18.5 (*n* = 89)	0.931	< 0.001	5.145	84.3	89.1
18.5 ≤ BMI < 24 (*n* = 1079)	0.915	< 0.001	4.345	83.5	84.2
24 ≤ BMI < 28 (*n* = 904)	0.901	< 0.001	4.115	81.0	84.2
BMI ≥ 28 (*n* = 415)	0.885	< 0.001	3.165	88.7	74.6
Total population (*n* = 2487)	0.899	< 0.001	4.405	78.7	85.6

*AMH* Anti-Müllerian hormone, *BMI* Body mass index, *AUC* Area under the curve

To distinguish women with PCOS from control, the AUC of the ROC curve for AMH was 0.899 in the unstratified population, while the AUCs when BMI was taken into account were 0.931, 0.915, 0.901, and 0.885 in the underweight, normal weight, overweight, and obese groups, respectively (Fig. 2). Based on the Youden index analysis, the optimistic AMH cut-off value was 4.405 ng/ml in the total population, with a sensitivity of 78.7% and specificity of 85.6%. In the group with BMI < 18.5 kg/m^2^, the optimistic AMH cut-off value was 5.145 ng/mL with a sensitivity of 84.3% and specificity of 89.1%, whereas in the group with BMI ≥ 28 kg/m^2^, the optimistic AMH cut-off value was 3.165 ng/mL with a sensitivity of 88.7% and specificity of 74.6%. For the BMI range categories of 18.5–24 and 24.0–28 kg/m^2^, the optimistic AMH cut-off values were 4.345 ng/mL and 4.115 ng/mL, respectively. Therefore, we concluded that groups with lower weights tended to have significantly higher AMH thresholds. Thus, the obese group had the lowest AMH thresholds among the groups (Table 3).

Consistent with the PCOS group, the tendency that the group with lower weight corresponded to higher AMH cut-off values was also applicable in the PCOM group (Supplementary Table 1 and Supplementary Fig. 2). In the underweight group, the optimal AMH cut-off value was 4.3 ng/mL, with a sensitivity of 70.7% and specificity of 82.7%. In the BMI ≥ 28 kg/m^2^ group, the optimistic cut-off value was 3.155 ng/mL, with a sensitivity of 79.3% and a specificity of 74.6%. Optimal cut-off AMH values were 3.635 ng/mL and 3.73 ng/mL for the BMI range categories of 18.5–24 and 24.0–28 kg/m^2^, respectively. In the same BMI category, patients with PCOM had a lower AMH diagnosis threshold than PCOS (< 18.5 kg/m^2^, 5.145 vs. 4.3 ng/mL; 18.5–24 kg/m^2^, 4.345 vs. 3.635 ng/mL, 24.0–28 kg/m^2^, 4.115 vs. 3.73 ng/mL, ≥ 28 kg /m2, 3.165 vs. 3.155 ng/mL). These cut-off values were then tested in the validation dataset, and the results are shown in Supplementary Table 2.

### The distribution of AMH in PCOS based on different phenotypes and severity of ovulation disorders

Supplementary Table 3 shows the basic information on PCOS phenotypes, and AMH was significantly different among the PCOS phenotypes (*P* < *0.001*). Type A PCOS with all symptoms had the highest AMH levels, while type B PCOS without PCOM symptoms had the lowest AMH levels, and the difference was statistically significant.

According to the severity of ovulatory dysfunction, patients had the highest AMH levels in the menstrual cycle of ≥ 180 days (*n* = 431), followed by those with a menstrual cycle of < 180 days (*n* = 3344), and PCOM (*n* = 2879) had the lowest AMH level (*P* < *0.001*) (Supplementary Fig. 3).

## Discussion

This retrospective study suggests that AMH is a potential diagnostic indicator of PCOS, which is adversely associated with BMI. With increasing BMI, the cutoff value of AMH for diagnosing PCOS decreased gradually. Second, we found that the AMH cut-off value for diagnosing PCOS was significantly higher than that for diagnosing PCOM. Moreover, AMH levels were correlated with PCOS phenotypes and menstrual cycle days in patients with PCOS.

AMH can be influenced by many factors, such as age, body mass index, pregnancy, oral contraceptives, and race [16]. Previous researches found that a strong inverse correlation was found between serum AMH levels and BMIs in patients with PCOS and without PCOS [2, 12, 17, 18]. Furthermore, a negative correlation between BMI and AMH was observed in women in the late reproductive years [19] and in women with diminished ovarian reserve [20]. Vagios et al. developed a model combining AMH levels and body mass index, which had good performance in predicting the probability of ovulatory dysfunction disorders [13]. Meanwhile, our study suggested that the cut-off value of serum AMH for diagnosing PCOS is negatively correlated with BMI. Therefore, it is irrational to use AMH level as a single indicator to diagnose PCOS in all populations. The BMI-stratified anti-Müllerian hormone cut-off values can serve as an important reference for clinical diagnosis.

The possible biological mechanism of the inverse correlation between BMI and AMH may result from changes in adipocytokines in obese women. A few studies have reported a positive correlation between adiponectin and AMH, and a negative correlation between leptin and AMH. Serum adiponectin concentrations decline in obese women, which influences enzymes of steroid synthetic pathways and may have a direct effect on AMH production through the receptors in the granulosa cells [21]. Moreover, increased serum leptin in obesity suppressed anti-Mullerian hormone gene expression through the JAK2/STAT3 pathway [22]. AMH has racial differences. A study reported the independent effect of ethnicity on serum AMH concentrations and found that Chinese women have a significantly lower AMH level than their European counterparts from age 25 [23]. Based on previous studies suggesting that AMH concentration varies between women of different ethnic backgrounds [23, 24], we examined the optimal cutoff value of AMH concentration to diagnose polycystic ovarian syndrome in Chinese infertility women from a single center. Consistent with previous studies, the cut-off value of AMH for diagnosing PCOS was lower than that for Western women.

It has been well established that AMH levels in PCOS are correlated with AFC. No study has reported the correlation of AMH values for predicting PCOM and PCOS. A previous observational study demonstrated a higher AMH production per follicle in PCOS with oligo-anovulatory phenotypes than in PCOM, while there was no significant difference between the ovulatory phenotype PCOS and PCOM [25]. All PCOS patients in our study had ovulation disorders, and in the matched BMI group, the AMH cut-off values for distinguishing PCOM from controls were lower than the cut-off values for distinguishing PCOS from controls. This suggests that AMH levels might be related to ovulation disorders and that there are other reasons for the increase in AMH levels in PCOS patients, in addition to antral follicle count. Serum AMH concentration is stimulated by dysregulated endocrine factors, such as androgens, insulin, and LH, which are common in PCOS. Abnormal granulosa cell function is involved in the pathophysiology of ovulation disorders in PCOS. In vitro exposure to high levels of androgens can prevent the attenuation of AMH, cause dysregulation of the AMH/SMAD signaling pathway, and lead to abnormal ovulation [26]. However, in women with PCOM, granulosa cell dysfunction is too low to cause ovulation dysfunction [27]. However, further experiments are needed to explore the correlation between AMH and PCOS.

Our study has several strengths. First, our study had a large sample size, which makes the results more reliable. Second, based on the racial diversity of serum AMH, our study was the first to combine anti-Müllerian hormone and BMI to diagnose polycystic ovary syndrome and polycystic ovary morphology in Chinese women. Third, we found differences between AMH levels in the diagnosis of PCOS and PCOM, which may promote the refined application of AMH in clinical practice.

The study had limitations. First, because of the large sample size, multiple doctors performed the ultrasound, but each doctor was uniformly trained and experienced. Although deviations in follicle counts were inevitable, they were evenly distributed among all the patients. Second, the diagnostic criteria for PCOM used in our study are Rotterdam criteria, which defines a cut off of 12 follicles to determine PCOM lower than the cut off of 25 follicles in the updated definition of PCOM in 2014 [28]. Compared to the early machines used when the Rotterdam standard was established, the resolution of modern ultrasonic equipment has improved. These may lead to an increase in the detection rate of PCOM in this study. Finally, this study was a single-center study, and a multicenter, prospective study is needed to further explore the value of AMH in the diagnosis of PCOS.

## Conclusions

In summary, our study demonstrated the refined application of AMH in clinical practice. First, AMH combined with BMI can provide an objective indicator for the individualized diagnosis of PCOS. Next, we found that the AMH cutoff value for diagnosing PCOS was significantly higher than that for diagnosing PCOM, and AMH could be used as an indicator of the severity of the PCOS phenotype. These findings contribute to our understanding of the role of AMH in reproductive physiology.

## Supplementary Information

**Additional file 1: Supplementary Figure1. **The serum AMHconcentrations in the population with PCOS and PCOM and the control group basedon different BMIs. PCOS,polycystic ovary syndrome; PCOM, polycystic ovary morphology; AMH, anti-Müllerian hormone; BMI, body mass index.

**Additional file 2: Supplementary Figure 2. **ROC curves for thediagnosis of PCOM based on different BMIs. BMI, body mass index; AUC,area under the curve.

**Additional file 3: Supplementary Figure3. **The serum AMHconcentrations in the PCOS and the PCOM groups based on the length of menstrualcycle. PCOS, polycystic ovary syndrome; PCOM, polycystic ovary morphology; AMH,anti-Müllerian hormone. PCOS (<180): patients with PCOS with menstrual cyclelength <180 days; PCOS (≥180): patients with PCOS with menstrual cyclelength ≥180 days.

**Additional file 4: Supplementary Table1.** Results from the ROC analysis for thediagnosis of PCOM based on different BMIs.

**Additional file 5: Supplementary Table2.** Validation of the optimal cut-off value in the validation data set.

**Additional file 6: Supplementary Table 3.** Basicclinical characteristics of the phenotype of PCOS.

## Data Availability

The data underlying this article cannot be shared publicly because of the privacy of individuals who participated in the study. The data will be shared upon reasonable request with the corresponding author.

## Abbreviations

AMH: Anti-Müllerian hormone
BMI: Body mass index
PCOS: Polycystic ovary syndrome
PCOM: Polycystic ovary morphology
ORs: Odds ratios
CIs: Confidence intervals
ROC: Receiver operating characteristic
ART: Assisted reproductive technology
AFC: Antral follicle count
FSH: Follicle stimulating hormone
LH: Luteinizing hormone
T: Testosterone
E2: Estrogen
P: Progesterone
AUC: Area under the curve
ANOVA: Analysis of variance
