# Supplementary figures and images for: The reference value of anti-Müllerian hormone to diagnose polycystic ovary syndrome is inversely associated with BMI: a retrospective study
Source: Reprod Biol Endocrinol. 2023 Feb 1;21:15. doi: 10.1186/s12958-023-01064-y (PMC9890853; doi:10.1186/s12958-023-01064-y)

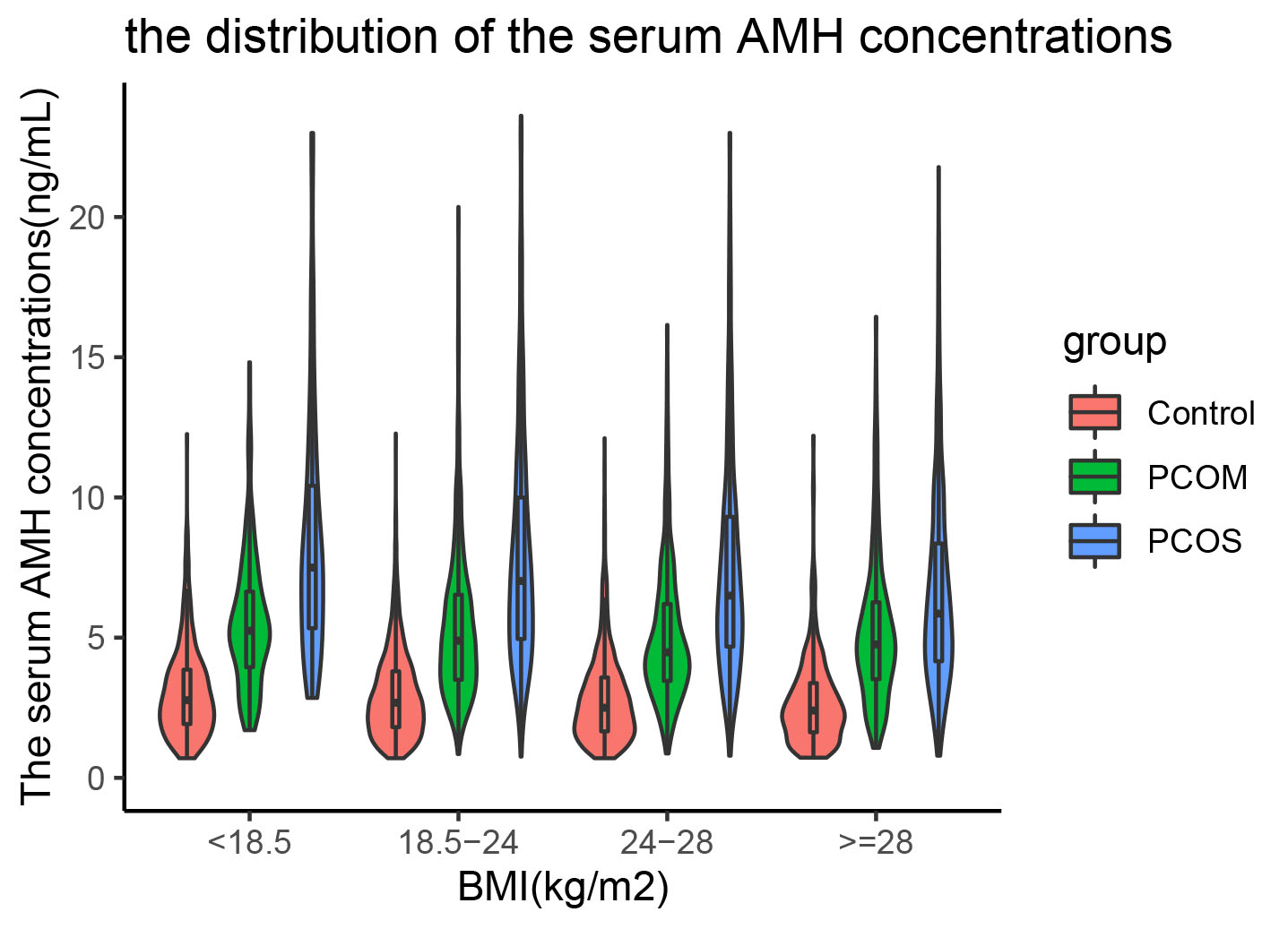

Supplement: Supplementary file 1 — Additional file 1: Supplementary Figure1. The serum AMHconcentrations in the population with PCOS and PCOM and the control group basedon different BMIs. PCOS,polycystic ovary syndrome; PCOM, polycystic ovary morphology; AMH, anti-Müllerian hormone; BMI, body mass index. [file 12958_2023_1064_MOESM1_ESM.jpg]

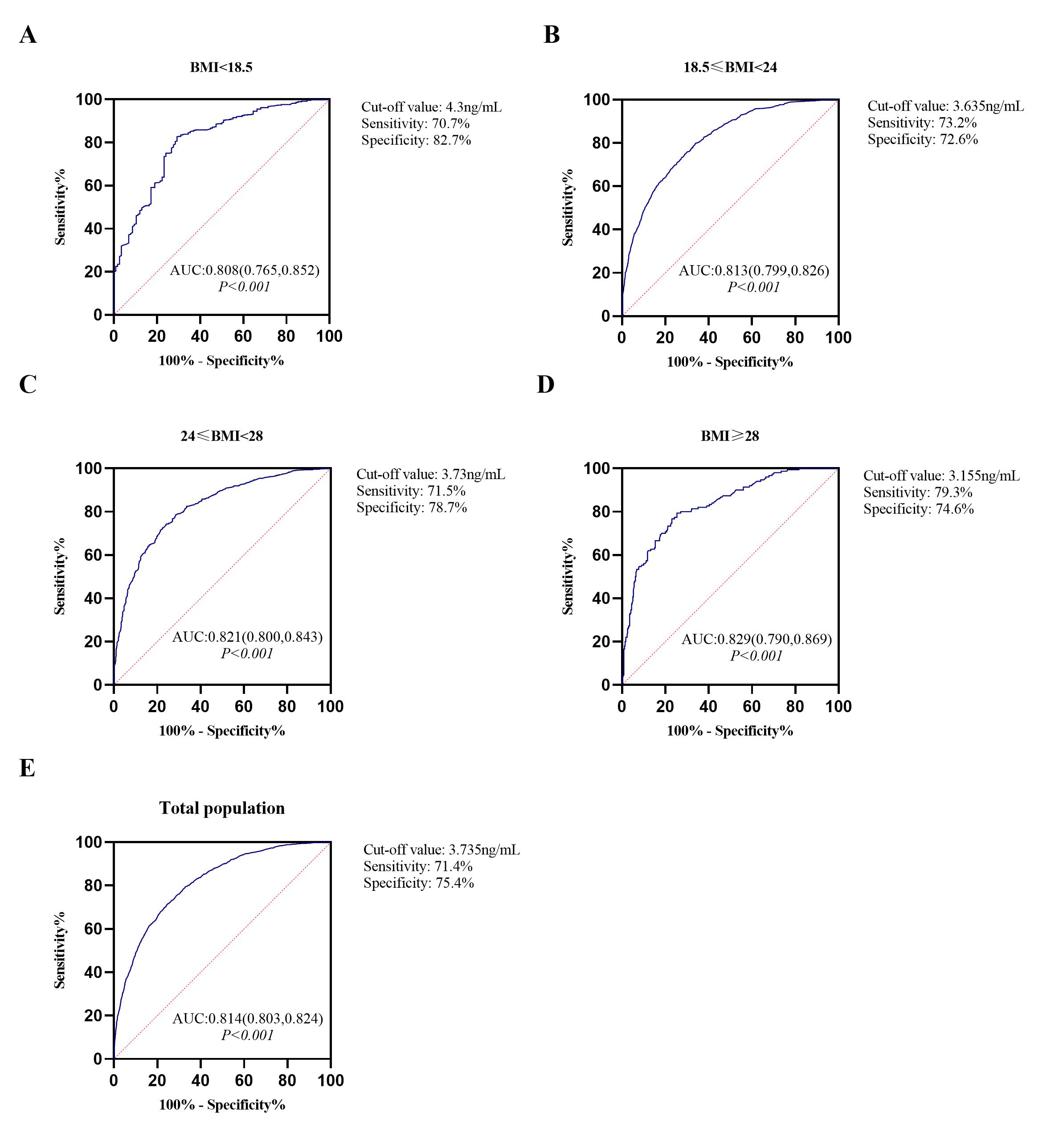

Supplement: Supplementary file 2 — Additional file 2: Supplementary Figure 2. ROC curves for thediagnosis of PCOM based on different BMIs. BMI, body mass index; AUC,area under the curve. [file 12958_2023_1064_MOESM2_ESM.jpg]

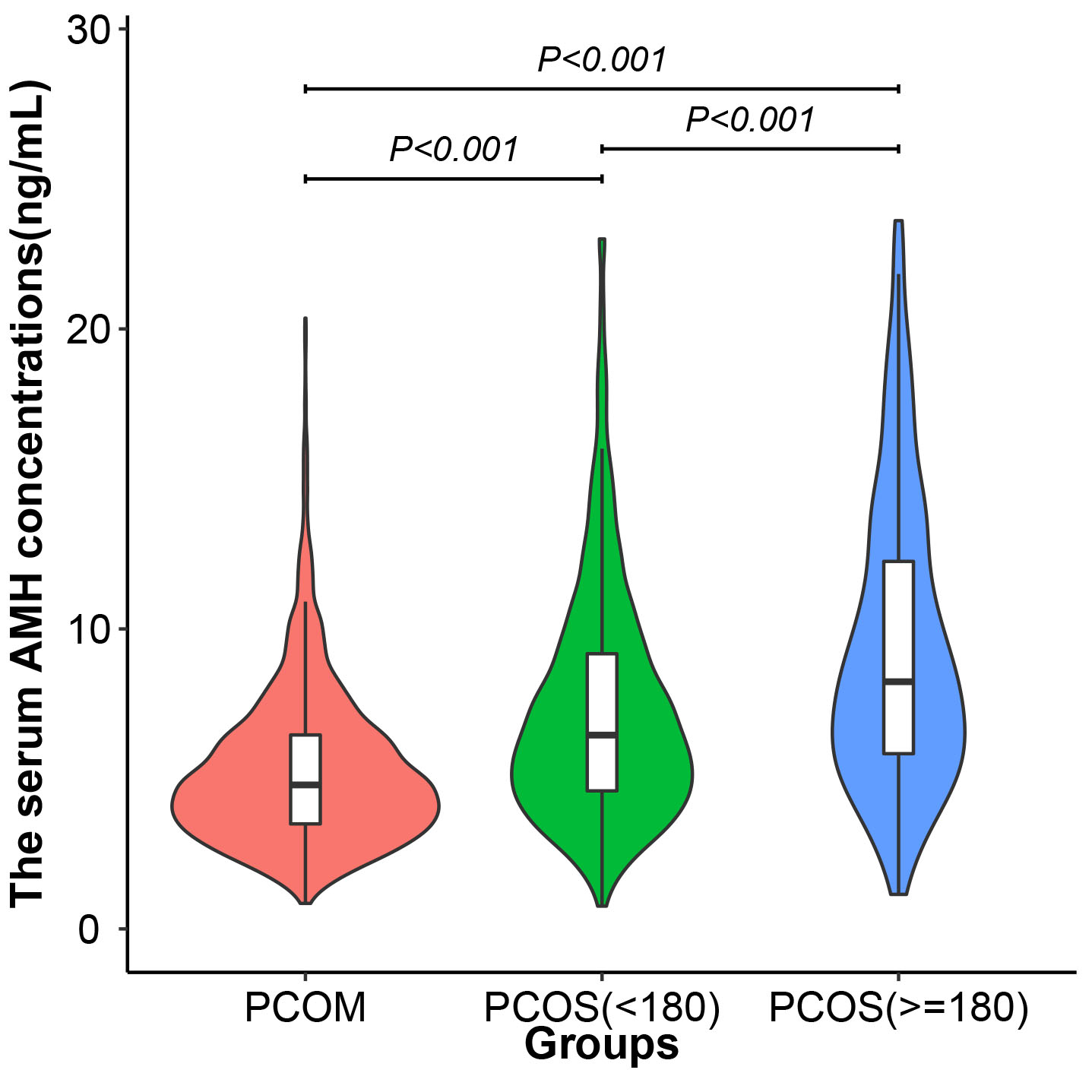

Supplement: Supplementary file 3 — Additional file 3: Supplementary Figure3. The serum AMHconcentrations in the PCOS and the PCOM groups based on the length of menstrualcycle. PCOS, polycystic ovary syndrome; PCOM, polycystic ovary morphology; AMH,anti-Müllerian hormone. PCOS (<180): patients with PCOS with menstrual cyclelength <180 days; PCOS (≥180): patients with PCOS with menstrual cyclelength ≥180 days. [file 12958_2023_1064_MOESM3_ESM.jpg]
