# Supplementary material for: The reference value of anti-Müllerian hormone to diagnose polycystic ovary syndrome is inversely associated with BMI: a retrospective study
Source: Reprod Biol Endocrinol. 2023 Feb 1;21:15. doi: 10.1186/s12958-023-01064-y (PMC9890853; doi:10.1186/s12958-023-01064-y)
Supplement: Supplementary file 4 — Additional file 4: Supplementary Table1. Results from the ROC analysis for thediagnosis of PCOM based on different BMIs. [file 12958_2023_1064_MOESM4_ESM.docx]

**Supplementary Table 1.** **Results from the ROC analysis for the diagnosis of PCOM based on different BMIs**

| **BMI Groups** | **AUC** | ***P* value** | **The optimal cut-off value of AMH (ng/ml)** | **Sensitivity (%)** | **Specificity (%)** |
| --- | --- | --- | --- | --- | --- |
| BMI<18.5  (n=116) | 0.808 | <0.001 | 4.3 | 70.7 | 82.7 |
| 18.5≤BMI<24  (n=1177) | 0.813 | <0.001 | 3.635 | 73.2 | 72.6 |
| 24≤BMI<28  (n=470) | 0.821 | <0.001 | 3.73 | 71.5 | 78.7 |
| BMI≥28  (n=150) | 0.829 | <0.001 | 3.155 | 79.3 | 74.6 |
| Total population  (n=1913) | 0.814 | <0.001 | 3.735 | 71.4 | 75.4 |

AMH, anti-Müllerian hormone; BMI, body mass index; AUC, area under the curve.
