# Supplementary material for: The reference value of anti-Müllerian hormone to diagnose polycystic ovary syndrome is inversely associated with BMI: a retrospective study
Source: Reprod Biol Endocrinol. 2023 Feb 1;21:15. doi: 10.1186/s12958-023-01064-y (PMC9890853; doi:10.1186/s12958-023-01064-y)
Supplement: Supplementary file 5 — Additional file 5: Supplementary Table2. Validation of the optimal cut-off value in the validation data set. [file 12958_2023_1064_MOESM5_ESM.docx]

**Supplementary Table 2.** **Validation of the optimal cut-off value in the validation data set.**

| **Groups** | **BMI (kg/m^2^)** | **The optimal cut-off value of AMH (ng/mL)** | **Sensitivity (%)** | **Specificity (%)** |
| --- | --- | --- | --- | --- |
| PCOS | <18.5 | 5.145 | 70.8 | 72.2 |
|  | 18.5-24 | 4.345 | 86.7 | 55.1 |
|  | 24-28 | 4.115 | 84.2 | 82.7 |
|  | ≥28 | 3.165 | 87.2 | 66.5 |
|  | total | 4.405 | 85.6 | 57.1 |
| PCOM | <18.5 | 4.3 | 73.5 | 81.8 |
|  | 18.5-24 | 3.635 | 73.2 | 70.3 |
|  | 24-28 | 3.73 | 64.4 | 78.2 |
|  | ≥28 | 3.155 | 83.3 | 66.5 |
|  | total | 3.735 | 69.9 | 74.0 |

PCOS, polycystic ovary syndrome; PCOM, polycystic ovary morphology; BMI, body mass index; AMH, anti-Müllerian hormone.
