# Supplementary material for: The reference value of anti-Müllerian hormone to diagnose polycystic ovary syndrome is inversely associated with BMI: a retrospective study
Source: Reprod Biol Endocrinol. 2023 Feb 1;21:15. doi: 10.1186/s12958-023-01064-y (PMC9890853; doi:10.1186/s12958-023-01064-y)
Supplement: Supplementary file 6 — Additional file 6: Supplementary Table 3. Basicclinical characteristics of the phenotype of PCOS. [file 12958_2023_1064_MOESM6_ESM.docx]

**Supplementary Table 3.** **Basic clinical characteristics of the phenotype of PCOS**

|  | **Phenotype A (n=2430)** | **Phenotype B (n=183)** | **Phenotype D (n=1162)** | ***P* value** |
| --- | --- | --- | --- | --- |
| Age (y) | 27.64 ± 3.05^3^ | 28.08 ± 3.32 | 28.24 ± 3.04^1^ | <0.001* |
| BMI (kg/m^2^) | 24.95 ± 3.58^3^ | 24.17 ± 3.70^3^ | 24.06 ± 3.53^2,3^ | <0.001* |
| T(ng/ml) | 2.37 ± 9.45^2,3^ | 4.72 ± 13.10^1,3^ | 0.31 ± 0.10^1,2^ | <0.001* |
| AMH (ng/ml) | 9.23 ± 4.63^2,3^ | 4.43 ± 2.85^1,3^ | 6.99 ± 3.42^1,2^ | <0.001* |
| LH/FSH | 2.18 ± 1.24^2,3^ | 1.20 ± 1.02^1,3^ | 1.47 ± 0.97^1,2^ | <0.001* |

PCOS, polycystic ovary syndrome; BMI, body mass index; T, testosterone; AMH, anti-Müllerian hormone; FSH, follicle stimulating hormone; LH, luteinizing hormone. Phenotype A: ovulatory dysfunction + hyperandrogenism + polycystic ovary morphology; Phenotype B: ovulatory dysfunction + hyperandrogenism; Phenotype D: ovulatory dysfunction + polycystic ovary morphology.
